# Gene Duplication, Translocation, and Molecular Evolution of *Dmrt1* and Related Sex-Determining Genes in Anurans

**DOI:** 10.3390/biom15091306

**Published:** 2025-09-11

**Authors:** Sagar S. Shinde, Paris Veltsos, Wen-Juan Ma

**Affiliations:** Research Group of Ecology, Evolution and Genetics, Biology Department, Vrije Universiteit Brussel, 1050 Brussels, Belgium; paris.veltsos@vub.be

**Keywords:** sex determination, master sex-determining genes, gene duplication, gene translocation, sex chromosome turnover, purifying selection, anuran, *Dmrt1*

## Abstract

Sex determination, the developmental process that directs embryos toward male or female fates, is controlled by master sex-determining genes whose origins and evolutionary dynamics remain poorly understood outside of a few model systems. In contrast to the highly differentiated sex chromosomes of mammals, birds, and *Drosophila*, most anurans (frogs and toads) maintain homomorphic sex chromosomes that exhibit a rapid turnover, even among closely related species. Master sex-determining genes evolve via gene duplication or via allelic diversification, and sex chromosome turnover is driven by gene translocation or novel mutations in the existing genes involved in the sexual developmental pathway. To uncover the mechanisms underlying the emergence of master sex-determining genes and sex chromosome turnover, we analyzed 53 published anuran genomes and one caecilian genome (>200 Mya divergence) and available transcriptomes. We asked how often master sex-determining genes arise by gene duplication, whether and how often gene translocation associates with sex chromosome turnover, and if master sex-determining genes evolve under positive selection. We find that chromosome-level synteny is remarkably conserved, with only a few fusions or fissions and no evidence for translocation of four candidate master sex-determining genes (*Dmrt1*, *Foxl2*, *Bod1l*, and *Sox3*). Only *Dmrt1* duplicated in 3 out of 50 species (excluding tetraploid *Xenopus*), and it showed strong testis-biased expression in all 8 species with available gonadal expression data. While *Dmrt1* has evolved under purifying selection, *Dmrt1* duplicates exhibit elevated nonsynonymous substitution rates and tendency towards positive selection. Lineage-specific amino acid changes were observed in the conserved DM domain of *Dmrt1*. These results demonstrate that, in anurans, master sex-determining genes rarely arise via gene duplication, and more likely evolve via allelic diversification. Sex chromosome turnover is not associated with gene translocation and is more likely driven by mutations on genes involved in sexual developmental pathways. All candidate sex-determining genes were under strong purifying selection, with the exception of duplications which are linked to positive selection. Our results suggest future research on anuran sex determination and sex chromosome evolution should focus on identifying allelic diversification and novel mutations on genes involved in sexual developmental pathways.

## 1. Introduction

Sex determination is a fundamental developmental process in which an embryo commits to either the female or male developmental pathway upon receiving certain cues during early embryogenesis. Despite its ubiquitous distribution across the Tree of Life, a remarkable diversity in sex-determining mechanisms have evolved [1,2]. Sex can be determined via genetic, environmental factors, or an interaction between the two [2]. For genetic sex determination, in many cases, the master sex-determining gene (acting at the top of the sex-determining cascade) is located either on the Y chromosome for the male heterogametic sex chromosome system (XX females and XY males), or on the W chromosome for the female heterogametic system (ZW females and ZZ males). Additionally, genetic sex determination can also operate through mechanisms such as the ratio of X (sex) chromosomes to autosomes, as in *Drosophila*, or through polygenic architecture, which has been reported in various fish lineages [1,2]. The XY system is characteristic of mammals, the ZW system is found in all birds, and both systems are found in insects, fishes, amphibians, reptiles, and plants [3,4,5,6,7,8]. A well-known system is the UV sex-determining system in most algae, where sex is expressed in the haploid phase (unlike animals and plants where it is expressed in diploid cells) [9,10,11,12,13]. For environmental sex determination, environmental factors or conditions influence early embryogenesis towards the female or male pathway. Environmental factors can be external, like temperature, pH, food resources, photoperiod, or biological factors like social environment, or an interaction between the two; for example, food allocation can be influenced by competition [14,15,16]. Some organisms change sex sequentially over their lifetime, so that an adult of one sex can switch to the opposite sex depending on social conditions or age [17], but these cases will not be further discussed here. The most studied type of environmental sex determination is temperature-dependent sex determination in various turtle lineages, where the temperature at a crucial thermally sensitive embryonic stage determines sex [18,19]. The genetic mechanisms of sex determination, however, remain largely unknown across the Tree of Life [1,20,21,22].

For genetic sex determination, sex chromosomes have repeatedly and independently evolved numerous times across the Tree of Life, representing astonishing convergent evolution of large genomic regions [5,8,23,24]. In sharp contrast to the highly degenerated (where many functional genes are lost) sex chromosomes in mammals, most birds, and *Drosophila*, sex chromosomes are largely homomorphic (appear identical with microscopy) and have minimal degeneration in most amphibians, fishes, many reptiles, and flowering plants [8]. Two non-exclusive mechanisms have been proposed to explain homomorphic sex chromosome evolution, inspired by amphibian biology: (i) the fountain of youth theory, which states that occasional recombination between sex chromosomes occurs in sex-reversed XY females [25], and (ii) rapid sex chromosome turnover, where different autosomes in different closely related species rapidly evolve a sex-determining role so that there is no sufficient time for sex chromosome degeneration [26,27,28]. Rapid sex chromosome turnover has been identified in amphibians, fishes, reptiles, and flowering plants, and more are expected to be revealed with advancing and cost-effective genomic sequencing [26,28,29,30,31,32,33,34,35,36]. The underlying genetic mechanisms remain largely unknown, with the exception of gene translocation of master sex-determining genes in Takifugu pufferfish, salmonid fishes, and strawberries [34,36,37].

Anurans (frogs and toads) are excellent systems to address pressing questions on the genetic mechanism of sex determination, as well as rapid sex chromosome turnover. The majority (>75%) of all studied anurans possess homomorphic sex chromosomes with little degeneration, yet extensive sex chromosome degeneration has also evolved in certain anuran lineages [38]. In addition, there is frequent and rapid sex chromosome turnover in anurans [29,30], and there are documented cases of both genetic and non-genetic sex determination. Moreover, experimental manipulation during early development is easy in frogs due to their external fertilization [39,40].

*Dmrt1*, *Doublesex*, and *Mab3-related transcription factor* 1, are characterized by the presence of a DM domain, a DNA-binding motif with an unusual cysteine-rich zinc DNA binding motif [41]. *Dmrt1* is indispensable both for the maintenance of testis development and function and the suppression of female-determining pathways in males across various vertebrate groups [41,42,43,44]. Its crucial role in sex determination has been documented across vertebrates, where *Dmrt1* (or its paralog) has been identified as the master, or candidate master, sex-determining gene, including in medaka fish (*Oryzias latipes*), tropical clawed frogs (*Xenopus laevis*), chickens (*Gallus gallus*), and the red-eared sliding turtle (*Trachemys scripta*), among others (reviewed in [22]). The highly conserved role of *Dmrt1* across vertebrates highlights its fundamental importance in the genetic regulatory networks that govern sex determination and gonadal sex differentiation.

In anurans, the genetic basis of sex determination is only known for the African clawed frog *Xenopus laevis*, where a dominant W chromosome duplication of *Dmrt1* (*DmW*) determines ovarian development [45]. Due to ancestral allopolyploidization, *X. laevis* possesses two copies of *Dmrt1*, *Dmrt1.L* (*DMRT1α*) and *Dmrt1.S* (*DMRT1β*), on autosomes 2L and 2S, respectively [45,46]. Partial duplication of *Dmrt1.S* (*Dmrt1β*) generated *DmW*, which maintains high similarity in the 5’-coding region (exons 1–4) but lacks the 3’-coding part which includes a transactivation domain-coding region (exons 5) [45,46,47,48,49,50]. The *Dmrt1* duplication occurs approximately 47 Mya after allotetraploidization, and *DmW* is found in several closely related *Xenopus* species. Other than *X. laevis*, *DmW* has been amplified in several females but none of the males in using targeted next-generation sequencing, suggesting it is female-specific in *X. gilli* while it is not consistently female-specific in most other *Xenopus* species [49,50]. *DmW* is required for ovary development in *X. laevis* females, and it functions as an antagonistic competitor of *Dmrt1* [48]. Both *Dmrt1* and *DmW* bind to the same regulatory regions of target genes (yet to be discovered) that are crucial in testis development. During embryonic gonad development, RNA of *DmW* is more abundant than *Dmrt1* in the primordial gonads of ZW tadpoles. This is thought to suppress the male-determining pathway because the *DmW* protein has the same binding site as the *Dmrt1* protein, but not its enhancer activity, thereby acting as a transcriptional repressor. The resulting failure of testis formation leads to ovary formation [45,48].

Beyond *X. laevis* in Pipidae, *Dmrt1* has been identified as the candidate master sex-determining gene in Hylidae and Ranidae. In four species of the *Hyla arborea* clade, a small sex-determining region including *Dmrt1* has been identified, with polymorphism in *Dmrt1* perfectly associating with phenotypic sex [51]. In *Rana temporaria*, a homologous region has the strongest F_ST_ difference between sexes and *Dmrt1* haplotypes perfectly correlate with male testis development in two populations [39]. Beyond *Dmrt1*, the *Bod1l* gene (*biorientation of chromosomes in cell division 1 like 1*) was recently identified as a candidate master sex-determining gene in *Bufo varilis* because it was the only region with homozygosity in females and heterozygosity in males [52]. The ‘usual suspect’ sex-determining genes refers to genes that fulfill essential functions during sexual development and are thus tightly linked to and have been repurposed to control sex determination. Two additional ‘usual suspect’ candidate sex-determining genes are *Foxl2* (*forkhead box protein L2*) and *Sox3* (*SRY-box transcription factor 3*) in anurans. *Sox3* was involved in sex chromosome system transitions (XY and ZW) in *Glandirana rugosa* and determine sex in several medaka (*Oryzias*) species [33,53]. *Foxl2* codes for a transcription factor essential for ovarian development and is suggested to suppress testis formation and was suggested to determine sex in tilapia and zebrafish [54,55].

Master sex-determining genes typically evolve in two ways: via gene duplication or allelic diversification. Both sex-determining genes of medaka fish (*DmY*) and the African clawed frog (*DmW*) have evolved by gene duplication from *Dmrt1* [45,56,57]. In both cases positive selection seems to have affected the duplicated genes, and in particular various amino acid mutations enhanced the binding affinity of their DM domain to DNA [58]. Beyond the two species and across anurans, it is unclear how common the mechanism of gene duplication is for the evolution of master sex-determining genes. However, testing allelic diversification requires well-phased assemblies of the X/Z and Y/W chromosomes, which remain technically challenging, and no suitable datasets are currently available to directly evaluate this hypothesis. Furthermore, population genetic theory predicts that X (or Z) chromosomes could play disproportionate roles in speciation and evolutionary divergence and predicts that X- or Z-linked divergence exceeds that on autosomes (the Faster-X effect) [59,60]. New master sex-determining genes are the first to be sex-linked and are therefore predicted to evolve faster than autosomal genes [61,62]. Positive selection on newly evolved master sex-determining genes has not been tested in lineages with rapid sex chromosome turnover, where master sex-determining genes rapidly evolve in the new sex chromosomes.

A previous study found at least 13 sex chromosome turnovers occurring within 50 Mya divergence in 28 Ranidae true frogs. Five chromosomes (Chr01, Chr02, Chr03, Chr05, and Chr08, based on the reference genome of *Xenopus tropicalis*) were recruited as sex chromosomes repeatedly and in a non-random manner [29]. *Dmrt1* on Chr01 has been identified as a candidate master sex-determining gene in *Hyla arborea* clades and *R. temporaria*, and Chr01 has been identified as determining sex in seven additional true frogs [29,39,51]. *Sox3* on Chr08 has been reported as a candidate master sex-determining gene in *G. rugosa* [53]. Chr05 in frogs harbors *Foxl2*, which is also one of the most important genes in the vertebrate sex-determination cascade [63]. It codes for a transcription factor essential for ovarian development and has also been implicated in the suppression of testis formation. *Foxl2* interacts directly with *Dmrt1*. The male-determining *Dmrt1* allele blocks the expression of *Foxl2* and in turn the development of ovaries, thus producing males [63,64]. Both gene translocation and novel mutations occurring in genes involved in sexual developmental pathways are proposed to drive sex chromosome turnover in true frogs, which remain to be tested with empirical data.

In this study, we utilize published whole genome assemblies and RNAseq datasets across anurans, to address to what extent gene duplication drives master sex-determining gene evolution. We further investigate the extent to which gene translocations drive sex chromosome turnover in anurans. We also analyze the evolution and selection affecting the ‘usual suspect’ sex-determining genes in anurans. In particular, whether the status of being (candidate) master sex-determining genes, or located on sex chromosomes, affects the selection they experience. Finally, we also analyze the conserved DM domain of *Dmrt1* across anurans, whether mutation patterns are lineage-specific and the association with functional divergence or adaptation across different frog species, and discuss how these changes might be associated with transcriptional control of downstream genes in the sex-determining pathway.

## 2. Materials and Methods

### 2.1. Anuran Genome Retrieval and Quality Assessment

We obtained published whole genome assemblies of 53 anurans and one outgroup (two-lined caecilian *Rhinatrema bivittatum*) from the National Center of Biotechnology and Information (NCBI) (Table S1). Additionally, we acquired the raw genomic reads for those available on NCBI for the purpose of verification of gene duplication, as well as all available transcriptomic data (raw reads as well as transcriptomes) (Tables S2 and S3). We assessed the quality of the genome and transcriptome assemblies using Benchmarking Universal Single-Copy Ortholog (BUSCO (v5.4.5)) scores [65]. They were either already published (Table S4A) or calculated using the Tetrapoda ortholog library (v.odb10) with the flag -m (‘--mode’ sets the assessment MODE: genome, proteins, and transcriptome) for genome or transcriptome, respectively (Table S4A,B).

### 2.2. Phylogeny of Species and DM Domain Sequences Across Anurans

We generated a phylogeny for the 54 amphibians using the available genome-wide 307 markers from Portik et al. (2023) [66]. First, the available multiple sequence alignment data (54 species; Supplementary File S1) were obtained and aligned with –msaProgram in PRANK (v170427) and 1000 bootstraps implemented in Guidance2. Second, the best sequence evolution model was identified using ModelTest-NG (v0.2.0-dev_20220721) [67] with multiple concatenated marker sequence alignments as input. The best model --model HKY+I+G4 was selected based on the Bayesian Information Criterion score. Third, we generated maximum likelihood genome-wide trees using raxml-ng with 1000 bootstraps, employing the GTR+I+G4 model. Finally, the bootstrap-supported best tree from the raxml-ng (v1.2.0) output was visualized in FigTree (v1.4.2) (https://github.com/rambaut/figtree, accessed on 1 November 2024). However, for the phylogenetic tree of 12 anurans with chromosome-level genome assemblies and annotation, we obtained the phylogenetic tree from timetree.org and derived their associated divergence time in millions of years.

To further verify that the duplicated copy was a true duplication of *Dmrt1* and not from another *Dmrt* family member, we also conducted phylogenetic analysis for the conserved DNA-binding domain (DM) of *Dmrt1*, along with DM coding sequences of all other *Dmrt* genes (i.e., *Dmrt2*, *Dmrt3*, *Dmrt4*, *Dmrt5*, and *Dmrt6*) across anurans. We extracted the DM domain coding sequences from all *Dmrt* genes in 53 anurans, as well as the partially duplicated region of *Dmrt1* containing the DM domain. We conducted the phylogeny using the procedure described above.

A substitution saturation test is crucial for ensuring the validity of sequence data when conducting both selection analysis and phylogenetic interpretation. We thus further assessed the sequences for substitution saturation using the index described by Xia et al. [68], implemented in DAMBE (v7.3.2) [69]. The indexes of substitution saturation (Iss) and critical Iss (Iss.c), estimated for symmetrical and extremely asymmetrical trees, were reported for each group of species in our study. In all cases, we found Iss was significantly less than Iss.c, suggesting that the sequences in our study are not saturated.

### 2.3. Chromosome-Level Synteny Analysis Across Anuran Genomes

Twelve chromosome-level assemblies with genome annotations were used for synteny analysis (Table S4A). In addition to available genomic resources, we also considered species to provide broadly phylogenetic representation across anuran lineages. Overall, on average one species from each monophyletic group was included to ensure a comprehensive comparison of synteny, with *Xenopus tropicalis* as the reference genome. Across anuran lineages with >200 Myadivergence (https://timetree.org/), we performed pairwise genome-wide synteny analysis using MCScanX (v20221101) [70]. MCScanX was designed for detecting and analyzing synteny and collinearity in comparative genomics and allows identification of homologous chromosomal regions across multiple genomes or subgenomes, aligning these regions using genes as anchors. Default parameters were used to generate pairwise alignments of collinear blocks using MCScanX [70]. The detected homologous regions across pairwise genome comparisons were subsequently merged into a single dataset, and the synteny was visualized with TBtools-II (v2.315) [71].

### 2.4. Analyses on Gene Duplications of Dmrt1, Foxl2, Sox3 and Bod1l

Orthologs of *Dmrt1*, *Foxl2*, *Sox3*, and *Bod1l* were identified based on coding sequence similarity and micro-synteny analysis (see details below). Accurate detection of orthologs and duplicated copies was performed with a combination of high-quality genome assemblies and further validation using raw genomic reads.

For *Dmrt1*, we could directly retrieve itself and its orthologs’ coding sequences from NCBI for several species including *Xenopus tropicalis*, *Hyla sarda*, *Nanorana parkeri*, *Rana temporaria*, *Pseudophryne corroboree*, *Bufo gargarizans, Bufo bufo, Eleutherodactylus coqui*, and *Spea bombifrons*. When direct retrieval from NCBI was not possible, we performed BLASTn against the anuran genomes using the *Dmrt1* coding sequence of closely related species [72]. BLASTn results were manually examined to confirm that the estimated length of the open reading frame (ORF) was complete. For a few species such as *Bufo gargarizans, Bufo bufo, Eleutherodactylus coqui*, and *Hyla sarda*, instead of the standard five exons for *Dmrt1*, *Dmrt1* with six exons was computationally predicted in the genome annotation files, with the additional annotated exon coding for an additional 14 amino acids. No clear expression data were detected from RNAseq data for this additional exon, and conserved coding sequences in all other frog lineages did not align with it. Furthermore, we examined *Rana temporaria*, utilizing published transcriptome data from three different populations and five developmental stages (G23, G27, G31, G43, and G46) to determine the number of exons expressed [73,74,75]. We consistently observed the expression of five exons, which led us to focus further analysis on the conserved five exons across all species. Taken together, the extra annotated small exon of *Dmrt1* in a few anurans is most likely a bioinformatic error; gene prediction is imperfect and detailed gene structure often requires careful additional manual curation, which is especially crucial for large frog genomes (3–9.6 G) [76,77,78].

We then investigated gene duplications by identifying both whole exon and partial exon duplications, as well as complete gene duplications. These were further evaluated by analyzing available genomic raw read data. All *Dmrt1* duplications, either whole gene or partial, always involved additional chromosomes. We manually inspected ORFs for truncations within duplicated regions to determine whether the duplicated sequences contained premature stop codons. For the phylogenetic analysis, *Rhinatrema bivittatum* was used as the outgroup. The length of *Dmrt1* coding sequences and total gene lengths are described in Table S5. To inspect gene expression of both the original and duplicated copies of *Dmrt1*, we obtained RNA-seq data from those species with *Dmrt1* duplication if available on NCBI (Table S3). Furthermore, we also obtained RNA-seq data with at least one tissue from both sexes for any anuran from NCBI, which only retrieved 14 anurans (Table S6). RNA-seq reads were first mapped to the corresponding genome assemblies, or to a closely related reference genome when no species-specific assembly was available, using STAR (v2.7.11a) with default parameters [79]. Read quantification at the gene level was performed with HTSeq-count (v2.0.3) [80], using the gene annotation file if available and retaining only uniquely mapped reads. To validate and complement the results, we also performed gene expression analysis on the same RNA-seq datasets using Cufflinks (v2.2.1) [81]. In this analysis, transcript assembly and quantification were carried out based on the same genome assemblies/annotations, and expression levels were normalized as FPKM (fragments per kilobase of transcript per million mapped reads). We followed the same pipeline to detect gene duplications for *Foxl2* and *Sox3*. Previous examination of *Bod1l* has shown well preserved synteny and no evidence of gene duplications across anuran genome 54, so we did not analyze it here.

### 2.5. Analyses of Gene Translocation of Dmrt1, Foxl2, Sox3, and Bod1l

To detect possible gene translocation across various chromosomes, we investigated both micro-synteny and chromosome-level macro-synteny. For the *Dmrt1* micro-synteny analysis, 25 species with chromosome-level genome assemblies were used. Species with only scaffold or contig-level assemblies were excluded, as *Dmrt1* was often located on scaffolds with adjacent genes positioned separately, which made synteny inference quite challenging. We focused on verifying chromosome-level and gene-level synteny for *Dmrt1* and its five flanking genes on both upstream and downstream regions. Of the 25 species considered, genome annotations were available for 12 species, which were used for the genome-wide synteny analysis. Despite the availability of annotations, some species lacked complete annotations for the syntenic genes. In such cases, we performed BLAST (v2.2.26) search to verify gene presence. If a gene was not assembled, no strong BLAST hit was detected or the gene was entirely missing from the genome. Thirteen genomes lacked annotation data. To validate synteny for these chromosome-level assemblies, we performed a BLAST search using annotated gene sequences from other species available on NCBI. While the exact start and end positions were not always determined, we identified the highest BLAST hit and used it to infer gene location. We used the same pipeline to conduct micro-synteny analysis for *Bod1l*, *Sox3*, and *Foxl2.* Finally, the chromosome-level macro-synteny was conducted as part of genome-wide synteny (see details in the earlier section (Section 2.3)).

### 2.6. Molecular Evolution and Selection Analysis for Dmrt1, Foxl2, and Sox3

To investigate molecular evolution and selection type acting on candidate sex determining genes, we used three branch-specific models implemented in PAML (v4.9j) using the codeml program [82,83]. Selection inference was based on the *d_N_/d_S_* ratio (*ω*), following a maximum likelihood approach. We tested three branch-specific models M0 (null model) assuming a single *ω* for all species. Branch-neutral (alternative model) assumes the foreground species evolves under neutral evolution and branch-free (alternative model) estimates *ω* independently for the foreground and background species. The analysis included *Dmrt1* coding sequences and its paralogs from 54 species, including the duplicated *DmW* of *Dmrt1* in *X. laevis* and the duplicated *Dmrt1* copy *in Pelodytes ibericus*. The genome-wide phylogenetic tree was used for such analysis. The codeml program was executed using F1x4 and F3x4 codon frequency models to assess the signature of relaxation, or intensification of purifying selection or positive selection.

Additionally, to evaluate the impact on selection inference of the *Dmrt1* duplicated copy in *Pelodytes ibericus*, we compared the results with and without the duplication using the branch-test model. To further assess *ω* patterns, we calculated *ω* using the free-ratio model in codeml, which estimates *ω* independently for each branch. The *ω* values of the foreground branch from the branch-test model were compared with the results from the free-ratio *ω* analysis, which remained largely identical. Lastly, we used the PAML branch-site model to identify specific codon sites under positive selection. Finally, similar branch test analyses were performed for the other sex-determining genes *Foxl2*, *Sox3*, as well as one randomly selected transcription factor gene, *E2F transcription factor 1* (*E2F1*), for a subset of 12 species.

### 2.7. Evolution of Conserved DM Domain in Dmrt1

We aligned the amino-acid sequences from 53 anuran species using PRANK (v170427) [84], applying default settings. To evaluate alignment reliability, we performed 1000 bootstrap replicates with the PRANK --iterate option. All sequences were inspected to ensure they begin with the initiating methionine (M) residue. The final consensus alignment was visualized in Jalview (v2.11.5.0) (alignment viewer) [85]. We then mapped unique and clade-specific amino-acid substitutions onto the alignment and corresponding phylogenetic tree, manually verifying each site against the phylogeny to confirm its consistency and taxonomic specificity. The *Dmrt1* protein sequence similarity score was calculated and visualized for the whole *Dmrt1* gene as well as for the DM domain using Jalview.

## 3. Results

### 3.1. Strong Preservation of Chromosome-Level Synteny Across Anuran Genomes

**Figure 1:**
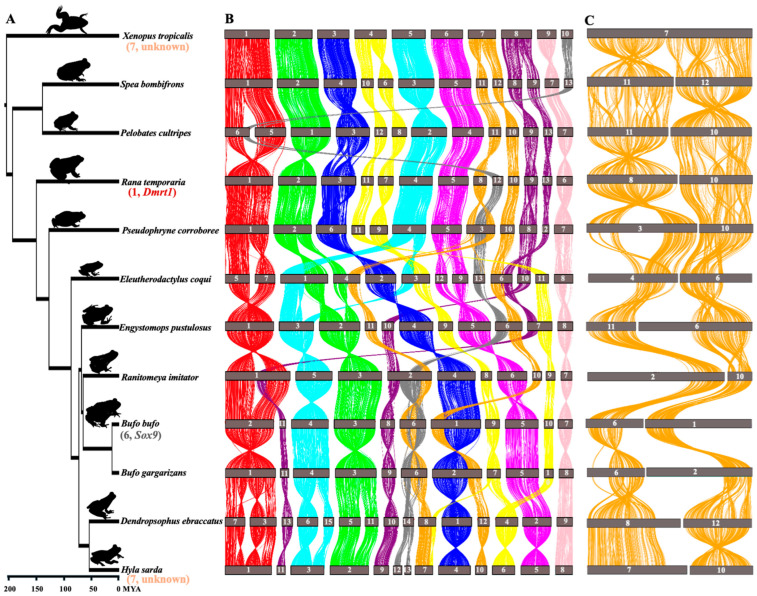
Genome-wide synteny across 12 anuran genomes with chromosome-level genome assemblies and genome annotation. (**A**) Phylogenetic tree obtained from timetree.org, with known sex chromosome or candidate master sex-determining genes indicated next to each species in parentheses. Colors are consistent with those of homologous chromosomes shown in the tree. (**B**) Chromosome-level synteny. Chromosomes are numbered and ordered by descending size (chromosome 1 is the largest, chromosome 14 is the smallest). (**C**) Example of chromosome 7, which is involved in sex determination in both *X. tropicalis* and *Hyla sarda* [86,87], which shows it has undergone fissions and fusions in various anuran lineages.

We obtained 54 amphibian genomes from NCBI—53 anurans and 1 two-lined caecilian (*Rhinatrema bivittatum*) which served as an outgroup (Table S1). Using *Xenopus tropicalis* as the reference genome, we inferred chromosome-level synteny, gene translocations, and duplications across the anuran genomes. All available (12) chromosome-level assemblies with genome annotations were used for synteny analysis. Assembly completeness was assessed using BUSCO with the tetrapoda_odb10 dataset (n = 5310) and resulting scores were >85%, with two exceptions (around 75%) (Table S4). The phylogenetic tree across the 12 anurans was generated from timetree.org, with estimated divergence time in millions of years (Figure 1A).

Genome-wide pairwise synteny was assessed based on sequence alignments and collinear blocks were generated using MCScanX and visualized in TBtools-II. The order of each pairwise comparison followed the smallest phylogenetic distance, starting with the reference genome *X. tropicalis* (Figure 1A). The chromosome-level synteny was well conserved across all anuran lineages, spanning >200 Mya divergence (Figure 1B). While there are several chromosome fissions and fusions in various frog lineages compared to the karyotype n = 10 of *X. tropicalis*, synteny blocks between these fission/fusion chromosomes were conserved (Figure 1B). For instance, *X. tropicalis* chromosome 7 is split into chromosomes 11 and 12 in *Spea bombifrons*, chromosomes 10 and 11 in *Pelobates cultripes*, chromosomes 8 and 10 in *Rana temporaria*, chromosomes 3 and 10 in *Pseudophrynes corroboree*, partial chromosomes 4 and 6 in *Eleutherodactylus coqui*, partial chromosome 6 and chromosome 11 in *Engystomops pustulosus*, partial chromosome 2 and chromosome 10 in *Ranitomeya imitator*, partial chromosomes 1 and 6 in *Bufo bufo*, partial chromosomes 2 and 6 in *B. gargarizans*, chromosomes 8 and 12 in *Dendeopsophus ebraccatus*, as well as chromosomes 7 and 10 in *Hyla sarda* (Figure 1C). Similarly, the synteny of *X. tropicalis* chromosome 1 corresponds to chromosomes 5 and 6 in *Pelobates cultripes*, or chromosomes 5 and 7 in *Eleutherodactylus coqui*, or chromosomes 3 and 7 in *Dendropsophus ebraccatus* (Figure 1B), suggesting independent chromosome fissions took place in various frog lineages. Large inversions were rare across highly diverged anuran genomes. The noticeable ones are two big inversions on chromosome 1 of *Hyla sarda*, corresponding to chromosomes 3 and 7 of *Dendropsophus ebraccatus* (Figure 1B), suggesting the inversions’ involvement in chromosomal fission in this species. Similarly, we identified local inversions across multiple chromosomes, such as chromosomes 4 and 5 between *B. bufo* and *B. gargarizans*, and chromosome 11 between *Spea bombifrons* and *Pelobates cultripes* (Figure 1B,C).

### 3.2. Dmrt1 Rarely Duplicated in Various Anuran Lineages with Fully Sequenced Genomes

Master sex-determining genes can evolve via gene duplication or allelic diversification. We first investigated whether *Dmrt1* was recruited as a master sex-determining gene via gene duplication in the anuran lineages with available whole genome sequences (Table S1). In addition to BLAST (based on coding sequencing of *Dmrt1*) searching, we also performed phylogenetic analyses of the DM domain across all *Dmrt* genes and their detected duplicated copies to provide a robust and thorough verification of all *Dmrt1* duplications across various anuran genomes. For the phylogeny of the DM domain, all duplicated copies largely clustered with *Dmrt1*, supporting their origin from *Dmrt1* duplication (Figure S1).

**Figure 2:**
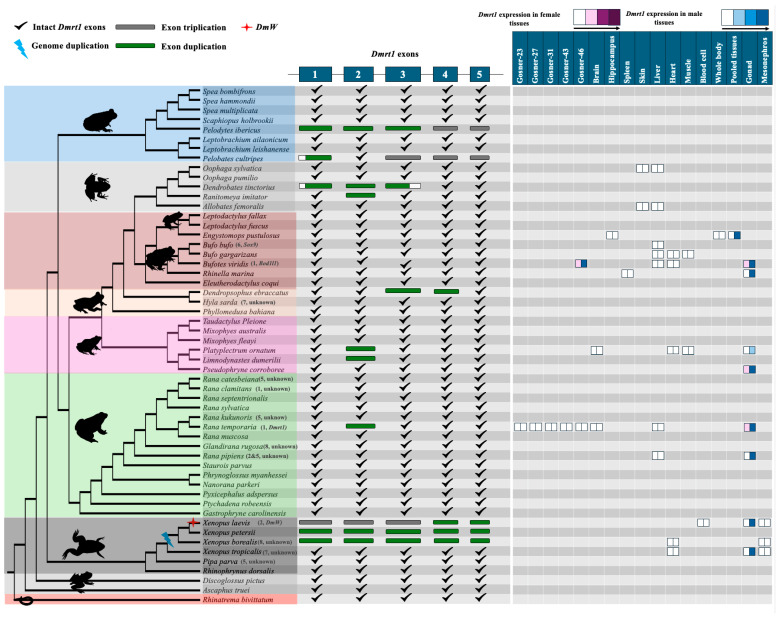
*Dmrt1* coding sequence structure and detected duplicated copies across 54 amphibia. The phylogenetic tree was conducted using multiple-loci alignment from Portik et al. (2023) [66]. *Dmrt1* exon duplications are color coded (2 copies in green, 3 copies in grey). Known sex chromosomes or candidate master sex-determining genes are indicated next to each species in parentheses. *Dmrt1* (the original copy) expression is plotted when data from both sexes with at least one tissue are available. The color gradient indicates expression in female (red) and male (blue) tissues. Color intensity indicates the scale of gene expression; the four expression bins are <5 FPKM, 5–10 FPKM, 10–25 FPKM, and >25 FPKM.

Across the 53 anurans, three were tetraploid (*X. laevis*, *X. petersii*, and *X. borealis*) which had two *Dmrt1* copies that arose via whole genome duplication. Additionally, *DmW* arose from duplication of *Dmrt1.S* (another copy is *Dmrt1.L*) and was recruited as a master sex-determining gene in *X. laevis* but was not consistently amplified in females in the other two species [49,50], which called into question *DmW*’s role as a sex-determining gene in two other *Xenopus* species. For the remaining 50 species, there were only three cases of near-complete gene duplication involving three (out of five) or more exons of *Dmrt1*, and five cases of duplication of only one or two *Dmrt1* exons scattered across the phylogeny (Figure 2). All duplications were verified using raw genomic sequencing reads (Table S2). Since the outgroup and ancestral branches of anuran lineages all have a single copy of *Dmrt1*, *Dmrt1* has probably undergone approximately eight independent duplication events (Table S7).

Near-complete duplication of *Dmrt1* was detected in three species (Figure 2). First, in *Pelodytes ibericus*, *Dmrt1* localized in the syntenic region on homologous chromosome 1 (of *X. tropicalis* reference genome), a complete duplicated copy resided on chromosome 10, and a third copy composed of only exons 4 and 5 was found on chromosome 6. Haplotype validation of the duplicated sequences was not possible due to lack of raw genomic reads. For the complete duplicated copy, the intact open reading frame was 305 amino acids; however, no transcriptomic data was available to detect gene expression levels. Second, one near-complete duplication of *Dmrt1* was identified in *Pelobates cultripes* (missing exon 2), and an additional third copy was identified composed of only exons 3, 4, and 5. All three copies, each with a distinct haplotype, were further supported by raw genomic reads (Table S2). The intact *Dmrt1* gene was located on chromosome 1, with the near-complete duplicated copy on chromosome 11 and the third copy on chromosome 14. The function of the truncated duplicated copies is unclear. While no premature stop codons were detected in both duplicated copies, there was no expression of either the near-intact or duplicated *Dmrt1* copies in tadpoles and adult tissues (Figures S2–S4). The conserved DM domain is primarily located on exon 1 and the beginning of exon 2, which are missing from the truncated duplicated copies. Third, in *Dendrobates tinctorius*, the duplicated copy was composed of a complete exon 2 and parts of exons 1 and 3. The two *Dmrt1* copies were located on two different scaffolds and could be distinguished from haplotype analysis using raw read data. Similarly, while there were no premature stop codons in the duplicated copy, transcriptomic data of skin, liver, gut, and brain tissues did not show expression of it (or *Dmrt1*) (Figures S5 and S6). The expression pattern is consistent with the strongly testis-biased or testis-restricted expression of *Dmrt1* observed across vertebrate lineages [22]. Determining whether the duplicated *Dmrt1* copies exhibit a similar expression profile will require additional RNA-seq datasets that include both somatic and gonadal tissues from both sexes. Only with such data will it be possible to infer the potential biological functions of the duplicated *Dmrt1* copies.

Approximately four independent duplications of one or two exons of *Dmrt1* have been detected in five frog species (Figure 2). Among them, is the European common frog *Rana temporaria*, one of the most studied anurans, where the *Dmrt1* gene is located within a syntenic region on chromosome 1 and has been identified as a candidate master sex-determining gene [39,40,88]. A complete copy of exon 2 was duplicated on chromosome 10, which was further supported by identification of two haplotypes using raw sequencing reads. The duplicated exon 2 was not expressed in any tissue (Figure S8). In another two species (*Platyplectrum ornatum* and *Limnodynastes dumerilii*) with duplicated copies of only exon 2, both copies were located on two different scaffolds. Due to low genome assembly quality, it remained unclear whether two copies were located on the same chromosome. We detected two different haplotypes for exon 2 in both species using the raw sequencing reads. In *Platyplectrum ornatum*, *Dmrt1* was extremely biased in male testes, with very low expression in ovaryies, and was not expressed in somatic tissues (Figure 2). It is possible that the duplication of exon 2 may have occurred in a common ancestor of the two species.

In *Dendropsophus ebraccatus*, duplication of exons 3 and 4 was identified. The intact exons are located on chromosome 3 within a syntenic region, while the duplicated exons are on chromosome 1, positioned close to each other. The assembled and annotated genome reveals that the duplicated copy of *Dmrt1* is located on a different chromosome compared to the syntenic copy of the gene. Both copies of the *Dmrt1* gene are identical, with no haplotype variation detected in either the raw genomic reads or the assembled genome. The pattern is consistent with a recent duplication event accompanied by minimal sequence divergence. Transcriptome data from whole-body tissue confirmed high expression of *Dmrt1* (Figure S9). In *Ranitomeya imitator*, duplication of exon 2 in *Dmrt1* was identified on chromosome 3, with the intact gene located on chromosome 1 within a conserved syntenic region. Two haplotypes were observed in the genome, but genomic raw data is not currently available for further verification. Transcriptome data from skin and brain tissues showed no expression of *Dmrt1* and duplicated regions in this species (Figures S10 and S11).

To investigate the sex/tissue-biased or sex/tissue-specific expression of the original *Dmrt1* copy, we analyzed all available RNAseq datasets containing data from both sexes, which are unevenly distributed among anurans. Although many datasets included only a single tissue, preventing formal statistical analysis, transcriptomic analysis consistently showed strong testis-biased or testis-specific expression, with minimal or no expression in ovaries, brain, liver, or other somatic tissues. Expression during developmental stages (Gosner stages 23, 27, 31, 43, and 46) was also very low or undetectable across 14 species, spanning diverse anuran lineages (Figure 2, Figures S7, S12 and S13; Table S6). The near testis-specific expression of *Dmrt1* across 14 distantly related anurans aligns with the established role of *Dmrt1* as a key regulator of testis development and potential sex determination, and its conservation across phylogenetically distant species underscores its evolutionary importance in male reproductive differentiation.

We also investigated gene duplication for two other strong candidate sex-determining genes in frogs—*Sox3* and *Foxl2*—and found no evidence. One possible duplication of *Foxl2* in *Rana muscosa* is likely an assembly error, as there was no sequence divergence between the two copies and no excessive genomic reads associated with the gene. Furthermore, *Bod1l* also showed no duplication across diverged anuran genomes [52]. Taken together, the current evidence suggests that *X. laevis* is a rare exception to the rule and master sex-determining genes do not commonly evolve in anurans via gene duplication.

### 3.3. No Evidence for Gene Translocation of Key Frog Sex-Determining Genes Driving Sex Chromosome Turnover in Frogs

**Figure 3:**
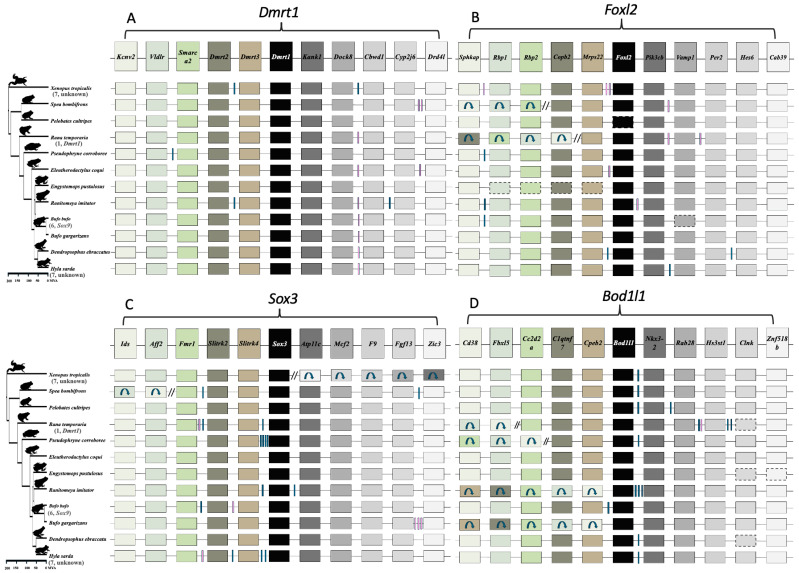
Gene order of the four candidate master sex-determining genes across the 12 anurans, and known sex chromosomes or candidate master sex-determining genes are indicated next to each species in parentheses. (**A**) *Dmrt1*, (**B**) *Foxl2*, (**C**) *Sox3*, and (**D**) *Bod1l* across 12 anurans with chromosome genome assemblies. The top row shows the focal gene, with its immediate upstream and downstream 5 genes based on the reference genome of *X. tropicalis*. Boxes with arrows indicate gene inversions, while paired tilted lines represent multiple genes with inverted orientations. Vertical blue boxes mark unique, uncharacterized LOC genes that are species specific, and pink lines denote other species-specific genes. Genes missing from the genome assembly are enclosed in dotted boxes. A detailed list of inversions and species-specific genes can be found in Tables S8–S11.

**Figure 4:**
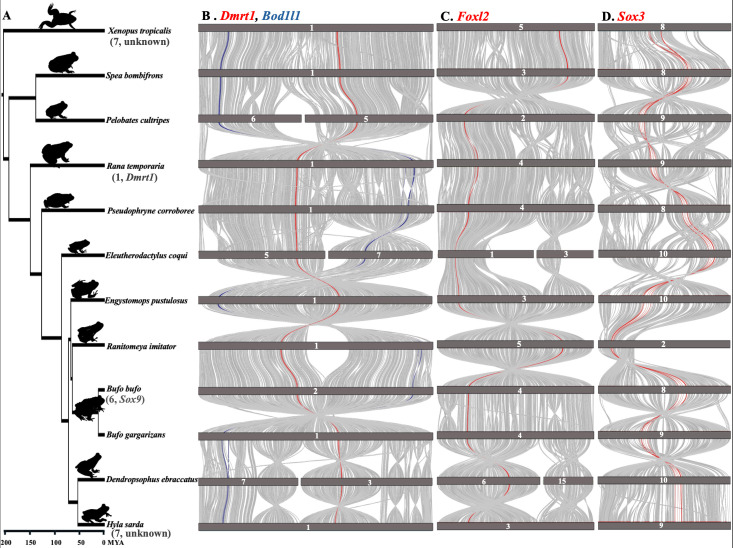
(**A**) The phylogenetic tree of 12 studied anurans alongside graphs of chromosome-level synteny for the micro-synteny region of (**B**) *Dmrt1* and *Bod1l*, (**C**) *Foxl2*, and (**D**) *Sox3.* Known sex chromosomes or candidate master sex-determining genes are indicated next to each species in parentheses.

We assessed whether gene translocation of the ‘usual suspect’ master sex-determining genes, *Dmrt1*, *Sox3*, *Foxl2*, *and Bol1l* drives sex chromosome turnover in frogs. We investigated both micro-synteny (consisting of five upstream and five downstream genes) and macro-synteny at the chromosome level across anuran genomes. For *Dmrt1*, the genomic regions flanking *Dmrt1* exhibit highly conserved synteny (Figure 3 and Figure 4, Table S8) within 12 anurans. Using the gene order from the reference genome of *X. tropicalis*, *Dmrt3*, *Dmrt2*, *Smarca2*, *Vldlr*, and *Kcnv2* were identified upstream, while *Kank1*, *Dock8*, *Cbwd3*, *Cytbp4502j6-like*, and *FoxD5a* were downstream (Figure 3A, Table S8). Various *Cytb* genes were annotated surrounding *Dmrt1* across anuran genomes (Figure 3A). In certain anurans, species-specific, uncharacterized *Loc* genes have been assembled in the syntenic region (Figure 3A, Table S8). In the outgroup *Rhinatrema bivittatum*, the upstream region contains five conserved genes. On the downstream region, the first three genes adjacent to *Dmrt1* (*Kank1*, *Dock8*, *Cbwd3*) are conserved, but the other two (*Cytbp4502j6-like* and D4 dopamine receptor-like genes) localize on different chromosomes. Instead, *Pgm5* and *Tmem252* are present. Overall, the *Dmrt1* micro-synteny inferred via a BLAST approach was largely conserved in 25 species with chromosome-level assemblies, and no local inversions were detected (Figure 3A and Figure 4). For other anuran genomes (28) with fragmented assemblies, while these *Dmrt1* flanking genes were generally located near each other, the fragmented assemblies did not allow to detect translocations within the synteny block.

Similar analysis of micro-synteny was conducted for *Foxl2*, *Sox3*, and *Bodl1l*, and it was also well conserved (Figure 3B–D, Tables S9–S11). In *Foxl2*, the overall gene order remained intact on both sides, except in *Rana temporaria*, where a local inversion on the downstream region, following the *Mrps22* gene, caused the last four genes to be rearranged in the syntenic region (Figure 3B and Figure 4C, Table S9). For *Sox3* in *X. tropicalis*, a local inversion on the upstream region caused the rearrangement of all five genes after *Sox3*, while the downstream region remained intact (Figure 3C and Figure 4D). Similarly, in *Spea bombifrons*, the last two syntenic genes on the downstream region were subject to local inversion, leading to a change in gene order. No local inversions were observed in other species for *Sox3* (Figure 3C and Figure 4D, Table S10). In addition, for *Bod1l* gene synteny, local inversions resulting in changes in gene order were detected on the downstream region, but the upstream region remained intact (Figure 3D and Figure 4B, Table S11). The chromosome-level macro-synteny of all ‘usual suspect’ candidate sex-determining genes was largely preserved across the various anuran genomes (Figure 1B and Figure 4).

A previous study found at least 13 sex chromosome turnovers in 28 Ranidea true frogs [29] and suggested that it could be driven by gene translocation, and/or novel mutations of genes involved in the sexual differentiation developmental pathway. *Dmrt1* and its paralogs have been suggested as candidate sex-determining genes in three frog species including *X. laevis*, *R. temporaria*, *H. arborea*, and chromosome 1, on which *Dmrt1* was identified as the sex chromosome in eight true frog species [29]. Both *Dmrt1* micro-synteny and chromosome 1 macro-synteny were strongly preserved in all 25 anurans with chromosome-level assemblies spanning > 200 Mya divergence (Figure 4, Table S8), suggesting that *Dmrt1* translocation is unlikely to have driven sex chromosome turnover in Ranidae, as well as across various anuran lineages. A similar result for *Foxl2*, *Sox3*, and *Bol1l*, suggests that repeated gene translocation is not the general genetic mechanism driving sex chromosome turnover across anurans.

### 3.4. Strong Purifying Selection Acting on Dmrt1 and Other Sex-Determining Genes

**Figure 5:**
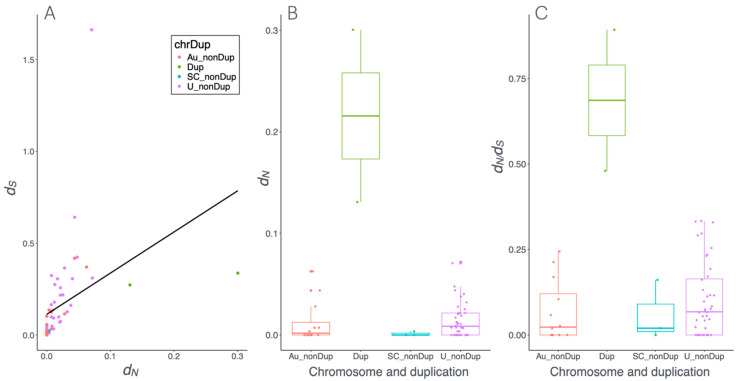
Plot of *d_N_* and *d_S_* values of *Dmrt1* and its duplicated copies for 53 anurans, including the status of sex chromosomes reported in the literature among 53 anurans. (**A**) Spearman’s correlation between *d_N_* and *d_S_* values. (**B**) Boxplot of *d_N_* values, (**C**) Boxplot of *d_S_* values across duplication and non-duplication status, with the latter also divided into autosomal, sex chromosome, and unknown. chrDup—chromosome and duplication status; Au—autosome; SC—sex chromosome; U—unknown; nonDUP—non-duplicated copy; Dup—duplication.

We investigated the molecular evolution of candidate master sex-determining genes *Dmrt1*, *Foxl2*, *Sox3*, *Bod1l* and their paralogs. The free-ratio models, implemented in PAML, were used to infer omega (*ω*, *d_N_/d_S_*) which is the ratio of non-synonymous (*d_N_*) to synonymous (*d_S_*) substitutions across 53 anurans (Figure 5 and Figure S14). If *ω* < 1 and smaller than background species, it suggests purifying selection. If *ω* = 1 the sequences evolve neutrally, while if *ω* > 1 the sequences may have evolved under positive selection. We first tested the global patterns of *d_N_/d_S_* along all branches of the phylogenetic tree (Figure S14). We identified two species, *Oophaga pumilio* and *Rana septentrionalis*, where *d_N_* exceeded *d_S_*, resulting in *ω* > 1. However, this is unlikely to have been the result of positive selection because there were very small *d_N_* (0.003556 and 0.007135) and there were even smaller *d_S_* values (0.000004 and 0.000007) (Table S12A,B). For the vast majority of anurans, *d_N_* was 0–0.05 and *d_S_* was 0.05–0.4, indicating purifying selection (Figure 5, Table S12A,B). In the case of the master sex-determining gene *DmW*, *ω* is 0.47, suggesting reduced purifying selection and tendency towards positive selection, while the autosomal (original) copy *Dmrt1* with a *ω* of 0.05 was under strong purifying selection.

We then performed a branch model test on all 53 anurans, including *DmW* and both duplicated copies of *Dmrt1* from three *Xenopus* species (*Dmrt1L* and *Dmrt1S*). By comparing the *ω* of the test species to that of the 52 background species, we detected varying selection pressures on *Dmrt1* or its paralogs. Among the test species, the ω of 17 species was greater than the background species and less than 1, suggesting reduced purifying selection. Statistically significant results were found in two species, for the duplicated copies of *Pelodytes ibericus* and *DmW* in *X. laevis* (Figure 5A,B, Table S12C). In contrast, the *ω* of *Rana septentrionalis* and *Oophaga pumilio* was greater than the background species and greater than 1, implying positive selection. However, this was not statistically significant, and likely the result of very low *d_N_* and even lower *d_S_* values for these species (Table S12C). For the remaining 37 species, *ω* was lower in the test than the background species and smaller than 1, indicating purifying selection (Figure 5A). Only *Discoglossus pictus*, *X. tropicalis*, and the outgroup *Rhinatrema bivittatum* showed statistically significant intensification of purifying selection (Table S12C). The *ω* of the duplicated copy of *Pelodytes ibericus* was 0.92 and appears to be under strong pressure towards positive selection.

We further used the branch-site model to identify which codon sites were under positive selection and assessed the distribution of sites in different selection schemes. Overall, more than 39 species showed that approximately more than 70% of sites were under purifying selection, while the remaining sites were under neutral selection. Only 14 species showed evidence of a few sites with positive selection with or without significance (Table S12D). Interestingly, *DmW* in *X. laevis* and the duplicated copy of *P. ibericus* showed multiple sites under positive section (Table S12D).

To investigate how different modes of selection influence the functional role of *Dmrt1* in master sex determination and sex linkage, we applied generalized linear models on all *d_N_* and *d_S_* values in the branch model. Overall, the vast majority of species showed very low values of *d_N_* and slightly higher values of *d_S_*, and the values were significantly positively correlated (Figure 5A, Spearman’s correlation, *ρ* = 0.74, *p* < 0.001), suggesting strong purifying selection. Furthermore, sex chromosome status does not affect selection, but gene duplication significantly affects ω (GLM: *ω* ~ chromosome + duplication, family = gamma, for sex chromosome: *p* = 0.27; for duplication, *p* = 0.002, Figure 5A). In particular, *d_N_*, *d_S_*_,_ and ω values were significantly higher for *Dmrt1* duplicated copies (*DmW* in *X. laevis* and the duplicated copy in *P. ibericus*, Figure 5B,C and Figure S15). For instance, in *Rana temporaria*, *Bufotes viridis*, *Rana clamitans*, *R. catesbeiana*, *and R. kukunoris*, when *Dmrt1* is located on sex chromosomes, the genes were under strong purifying selection but not significantly different than those on autosomes. Taken together, *Dmrt1* is under strong selective constraint overall. This could be due to its crucial role in testis formation, in meiosis [89], involvement of ovary function [90,91], or a combination of these [22].

Similarly, for the two other candidate sex-determining genes, *Foxl2* and *Sox3*, *d_N_/d_S_* analyses across anurans revealed that both *d_N_* and *d_S_* were small, and *d_S_* values were consistently higher than *d_N_* (Tables S13A and S14A). In terms of selection patterns, out of 53 analyzed anurans, more than 32 species exhibited strong purifying selection on both *Foxl2* and *Sox3* (Tables S13B and S14B). In the remaining species, a reduction in the strength of purifying selection was observed. While the variation in *ω* between background and foreground branches was generally minor, some species showed substantial differences, indicating a relaxation in purifying selection. Given that *Foxl2* and *Sox3* have not been clearly identified as candidate sex-determining genes across anurans, so GLM analysis on sex chromosome status was not conducted. Taken together, these results suggest that major candidate sex-determining genes are predominantly evolving under purifying selection across anurans, with occasional relaxation events in certain lineages.

### 3.5. Conservation and Lineage-Specific Mutations on the DM Domain

**Figure 6:**
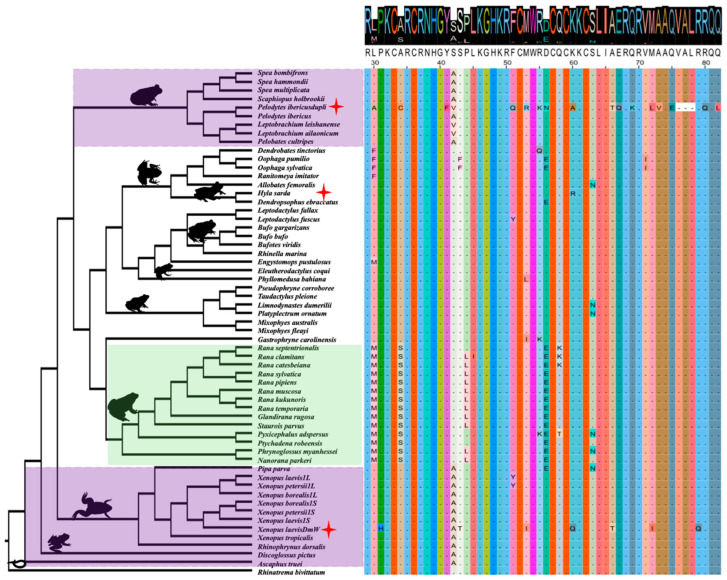
The protein sequence alignment and protein similarity analysis for the DM domain of *Dmrt1* across 53 anurans. In the top panel, colors indicate amino acid identity, while the size of each amino acid letter reflects sequence similarity across anurans, with larger letters denoting higher similarity. Sequence similarity was scored with an average of 0.96 (out of 1, with 1 representing 100% similarity without mutation), indicating high sequence similarity. Green shading highlights the Ranidae lineage with a lineage-specific amino acid mutation combination, and purple shading possibly indicates retention of ancestral amino acid in the basal lineages at position 42. Red star indicates certain amino acid mutation sites are under positive selection (position 64 in *H. sarda*, and various sites in two *Dmrt1* duplicated copies, *DmW* of *X. laevis* and *Pelodytes ibericus*).

The conserved DM domain of *Dmrt1* consists of 54 amino acids, spanning positions 29 to 82 of the *Dmrt1* protein on exons 1 and 2 (Figure 6). The DM domain is one of the most conserved motifs, reflected by the very high protein sequence similarity (0–1, with 1 representing 100% similarity), with an average 0.96 score compared to a whole *Dmrt1* average score of 0.83 (Figure 6, Figures S16 and S17). Despite the conserved DNA-binding function, we observed lineage-specific amino acid substitutions that suggest potential functional divergence or adaptation. First, within the DM domain, we identified unique Ranidae lineage-specific amino acid substitution combinations in 14 species of this lineage. These specific amino acid substitution combinations include a leucine (L) to methionine (M) change at position 30, an alanine (A) to serine (S) change at position 34, a proline (P) to leucine (L) change at position 44, and a D (aspartic acid) to E (glutamic acid) change at position 56 in nearly all 14 species (Figure 6, yellow highlight). Among these amino acid sites, codon site selection analysis suggested that mutations were not significantly under positive selection (Table S12D). Beyond the Ranidae group, four additional species *Oophaga pumilio*, *Oophaga sylvatica*, *Dendropsophus ebraccatus*, and *Pipa parva* also exhibited the D to E substitution at position 56, suggesting convergent evolution at this position. This convergence may reflect selective pressure on this residue. Possibly, these specific mutations modify the DNA-binding affinity or protein–protein interactions of the *Dmrt1* protein. The recurrence of these mutations specifically within Ranidae suggests possible lineage-specific functional tuning of *Dmrt1*, and the exact function of these substitutions’ combination and why this occurs in Ranidae is interesting and requires further investigation.

Another interesting finding is the at position 42 (purple highlight), 17 species at ancestral branches harbor an A (alanine) amino acid, two species have a valine (V), and the remaining species have a change from alanine (A) to serine (S). The parsimonious scenarios are (i) one mutation from S to A took place once in the ancestral branches, and three species had another round of two independent mutation events from S to V. This requires two mutation changes; (ii) in the derived lineages, one mutation from A to S took place, and three species had another found of two independent mutation events from A to V. Both scenarios require similar mutation changes, and it is unclear which event is most likely. Nevertheless, selection analysis on codon sites showed no significant positive selection at this position in all anurans. The repeated occurrence of the change suggests that certain sites within the DM domain may be subject to change, which could reflect the capability of DNA binding affinity and affect various pathways it interacts with.

Finally, in *H. sarda* (red star), we identified a site under positive selection in the DM domain at position 60, with an amino acid change from lysine (K) to arginine (R). Multiple sites (positions 43, 53, and 72) of the *Dmrt1* duplicated copy *DmW* in *X. laevis*, and the *Dmrt1* duplicated copy of *Pelodytes ibericus*, are under positive selection, although these are not statistically significant (Table S12D).

## 4. Discussion

Our aim is to investigate the genetic mechanisms underlying the emergence of new master sex-determining genes and the rapid turnover of sex chromosomes in anurans. Comparative genomic analysis revealed a highly conserved chromosome-level synteny across anuran genomes spanning > 200 million years divergence, with certain chromosomal fusions and fissions also preserving good collinearity. We further detected rare gene duplications of *Dmrt1* (3 out 50, excluding tetraploid *Xenopus* frogs), and no duplication was detected for *Foxl2*, *Bod1l*, and *Sox3*. *Dmrt1* showed a near testis-specific expression across all eight anurans with gonadal expression data. No gene translocation events were detected across anuran genomes for all four sex-determining genes. Selection analyses showed four genes were under purifying selection. For *Dmrt1*, the status of master sex-determining gene or sex linkage, did not affect the selection scheme, but gene duplications significantly contributed to positive selection. Finally, at the conserved DM domain region of *Dmrt1*, as an initial step, we described a few interesting cases of lineage-specific amino acid substitutions in Ranidea, ancestral branch specific substitution, and finally positive selection in one mutation in *H. sarda* and two *Dmrt1* duplications in *X. laevis* and *Pelodytes ibericus.*

Chromosome-level synteny is well preserved across anuran genomes, which is consistent with previous conclusions [92,93]. With the karyotype number varying between 10 and 14 across the 12 chromosome-level anuran genome assemblies, we detected several cases of chromosome fissions and fusions. However, genomic regions remain largely collinear at and surrounding the fission and fusion regions. For instance, between the genomes of *Eleutherodactylus coqui* (N = 10) and *Engystomops pustulosus* (N = 11), five chromosomal fusions and four additional fissions were detected. Yet all these surrounding regions remain largely colinear between the species. The evidence of conserved synteny across anurans despite chromosomal fusions and fissions suggests conserved chromosomal evolution shaping the evolution of recombination and gene order in anurans, which is also consistent with the slow genome evolution found recently [93]. Indeed, extreme heterochiasmy, where recombination is restricted to telomeric regions in males but distributes evenly across female genomes, has been documented in various anuran lineages with both XY and ZW sex chromosome systems (reviewed in [38]). Furthermore, the conserved synteny could also be a consequence of rapid sex chromosome turnover, where sex chromosomes frequently alternate among related frog species and across lineages [29]. Turnovers cause the whole genomic regions to constantly reshuffle, and recombination restores regularly, which may prevent the large structural variation and genome rearrangement spread and get fixed across anuran genomes.

New master sex-determining genes can evolve by gene duplication or allelic diversification [94]. The absence of well-phased X/Z and Y/W assemblies presents a major obstacle for testing the allelic diversification hypothesis. Therefore, in this study we primarily focus on evaluating the gene duplication hypothesis instead. The only known master sex-determining gene in anurans, *DmW*, evolved via gene duplication of *Dmrt1* in the African clawed frog *X. laevis* [45]. Rare duplications were detected in the ‘usual suspect’ master sex-determining genes, *Dmrt1*, *Foxl2*, *Sox3*, and *Bod1l* across 53 anuran genomes. In two species with near-complete *Dmrt1* gene duplication (*Pelodvtes ibericus* and *Dendrobates tinctorius*), the master sex-determining gene remains unknown, and further research is needed to determine whether the duplicated copy acquired a master sex-determining role. Our analysis suggests that new master sex-determining genes rarely evolve by gene duplication in anurans, the exception being *X. laevis*. Allelic diversification seems like a more likely mechanism for the evolution of novel genetic sex determination in anurans. One caveat of our analysis is that due to the difficulty in Y chromosome assembly, most chromosome-level genome assemblies are from the homomorphic sex (either XX female or ZZ male) (Table S1), we therefore cannot evaluate if the duplications occurred on the Y or W chromosome.

New master sex-determining genes have been found to evolve via allelic diversification in various teleost fish lineages, including the fighting fish (*Betta splendens*), sablefish (*Anoplopoma fimbria*), three Scatophagidae fish species, and the black carp (*Mylopharyngodon piceus*) [95,96,97]. In all these cases, different types of transposable elements were involved, and inserted in the promoter, or intronic, region on the *Dmrt1* Y copy to directly or indirectly increase *Dmrt1* Y copy expression. The only case of allelic diversification in anurans is the *Bod1l* gene in *Bufo viridis* [52]. It would be quite valuable to evaluate how common the allelic diversification mechanism is across anurans. This is not an easy task, as it would require phased X/Z and Y/W chromosome assemblies to identify the structural differences between the X/Z and Y/W copy.

Most anurans have highly conserved micro-synteny on the same locations across homologous chromosomes, with occasional local inversions, such as in *Foxl2*, *Sox3*, and *Bod1l*. Thus, no gene translocation was detected for the four top candidate sex-determining genes (*Dmrt1*, *Bod1l*, *Foxl2*, and *Sox3*) across various anuran genomes. Sex chromosome turnovers have been documented in many anurans, fishes, reptiles, insects and flowering plants [26,29,30,31,32,33,34], which can be driven by master sex-determining gene translocation, or novel mutations of genes involved in the sexual developmental pathway but took over a master sex-determining role [29]. Despite the wide spread of sex chromosome turnovers across animals and flowering plants, the underlying genetic mechanism is largely unknown. The only identified lineages are Takifugu fishes, salmonid fishes and strawberries, where master sex-determining genes from one species (repeatedly) translocated to another chromosome to determine sex, often involving certain types of transposable elements [32,34,36]. We did not detect any gene translocation in the four genes investigated here, suggesting gene translocation is not responsible for the rapid sex chromosome turnover across Ranidae, as well as across anurans. Therefore, novel mutations on genes involved in the sexual developmental pathway is a more probable mechanism and would need to be tested with comparative genomics, requiring whole genome assembly to identify the sex-determining regions and candidate genes.

Theory predicts that at least at the initial phase, a newly evolved master sex-determining gene is under somewhat positive selection and can sweep rapidly if it counterbalances a suboptimal sex ratio or resolves a sex-linked conflict [98,99]. In line with this prediction, both newly evolved master sex-determining genes *DmY* in medaka fish (*Oryzias latipes*) and *DmW* in the African clawed frogs (*X. laevis*) were under positive selection [56]. However, across 53 anurans, *Dmrt1* in the vast majority of them is under strong purifying selection, with very small values for both *d_N_* and *d_S_*. The strong purifying selection is also happening for those species where *Dmrt1* was identified as a candidate master sex-determining gene or was located on the sex chromosomes. Together this suggests *Dmrt1* is under strong evolutionary constraints and mutations cannot accumulate on this gene. This is consistent with *Dmrt1*’s conserved role in testis formation and development across various vertebrate groups [22]. Furthermore, pleiotropic effects can also contribute to its evolutionary constraint. *Dmrt1* was reported to be a molecular controller for meiosis entry, and also a requirement for ovary development in rabbits or certain fish [89]. Interestingly, the only two cases where genes tended towards positive selection and more mutations contributed to *d_N_* than *d_S_* were the duplicated copy of *Dmrt1* in *X. laevis* and *Pelodytes ibericus*. In the case of *DmW* in *X. laevis*, the original copy *Dmrt1* is still functional and required for testis formation and development, yet the duplicated copy *DmW* was free from selection constraint and was under positive selection. Although the role of the duplicated copy of *Dmrt1* in *Pelodytes ibericus* is unclear, a similar scenario where a duplicated copy freed itself from selection constraints and was under positive selection is possible, and the exact biological significance will require further molecular analyses.

The DM domain is a very conserved DNA-binding motif, shared by in total 9 *Dmrt* gene families across vertebrate lineages [22], of which three of them (*Dmrt7*, *Dmrt8*, and *Dmrt2b*) are either mammal- or fish-specific. Furthermore, this domain is homologous to *Doublesex (dsx)* in insects, which regulates sexual differentiation [100,101]. In mice, *Dmrt1* is necessary for testis maintenance and is sufficient to induce female-to-male cell fate reprogramming in vivo. *Dmrt1* expression in female ovary cells at around the sex-determining period could suppress *Foxl2* expression, which is at the top of the female-determining pathway. Overall, *Dmrt1* is in the crossroad of sex determination, acting as transcriptional factor, interacting with various other downstream genes to determine male or female pathways [95]. We detected several lineage-specific amino acid mutation combinations on the DM domain. One of the most striking ones is the Ranidae-specific M-S-L-E mutation combination occurring at the positions of *Dmrt1* proteins 30, 34, 44, and 56. Selection analysis showed these mutations were not under positive selection, but they could well be related to enhancing DNA binding affinity. Similarly, for *DmW* copy, H-T-I-Q-T-I-Q mutations were detected in the same DM protein region, most of them were not under positive selection, and the mutations are overall related to increased DNA binding affinity [58]. Another amino acid substitution is at position 42 in the most ancestral branch, where the amino acid A is harbored in the derived lineages with S. The current parsimonious scenarios both required two substitution change events, and it is unclear how the substitution changes and the functional explanation remains unclear. The next step would be to evaluate the functional consequences of these amino acid substitutions in a molecular context, for example by assessing their impact on DNA binding affinity, and to explore potential associations with broader life history traits and evolutionary changes.

## 5. Conclusions

To conclude, chromosome-level synteny is highly conserved across anuran genomes, and four candidate master sex-determining genes showed rare gene duplications or translocations. Furthermore, gene translocation is unlikely to be driving the frequent sex chromosome turnovers across anuran lineages. We therefore propose that new master sex-determining genes affecting sexual development pathways most likely evolve in anurans via allelic diversification, and the next step is to evaluate this mechanism across anurans. This would require (i) good chromosome-level genome assemblies to identify the sex-determining region, and (ii) phased X/Z and Y/W chromosome assemblies to detect diversifications and novel mutations of the master sex-determining gene. All candidate sex-determining genes are under strong purifying selection, regardless of their role as master sex-determining gene or in sex linkage. Our findings suggest that gene duplication may have released *Dmrt1* from selection constraints, facilitating the acquisition of novel adaptive functions under positive selection, as exemplified by *DmW* in *Xenopus laevis*. The positively selected sites identified in the duplicated *Dmrt1* copy of *Pelodytes ibericus* point to potential functional innovations, although their exact biological significance remains to be elucidated through further molecular analyses. Finally, there are interesting lineage-specific amino acid mutations in Ranidae but their potential role in enhancing DNA binding affinity or other function requires further investigation.

## Data Availability

All analyzed data are downloadable from NCBI. The scripts for analyzing all related results have been deposited in the GitHub repository: https://github.com/TheWMaLab/Duplication_evolution_MSDgenes_in_anurans, accessed on 1 November 2024.

## Supplementary Materials

The following supporting information can be downloaded at https://www.mdpi.com/article/10.3390/biom15091306/s1, Supplementary File S1: Multiple sequence alignment of 307 concatenated genome-wide markers from focal studied 54 amphibian species, based on data from Portik et al. (2023) [67]; Supplementary figures: Figure S1: Phylogenetic tree of the DM domain from all *Dmrt* genes across anurans. The tree illustrates the evolutionary relationships among DM domains. Red-colored tips indicate duplications of the *Dmrt1* DM domain. Figure S2: RNA-seq reads aligned to exons 1 to 5 of *Dmrt1* in *Pelobates cultripes* (Western spadefoot toad). Grey and white boxes represent reads aligned to the genome, showing coverage across the exons. All exons displayed very low expression, with exon 1 showing minute read coverage. The RNA-seq dataset, derived from whole-body tadpole samples (SRR7817206, SRR7817207, SRR7817208, SRR7817209, SRR7817210, SRR7817211, SRR7817212, SRR7817213, SRR7817214, SRR7817215, SRR7817219, and SRR7817220), was mapped to the reference genome (GCA_933207985.1_aPelCul1.1_chrom_genomic.fna). The locations of each exon are shown as BED records in the bottom row. Figure S3: RNA-seq reads aligned to the duplicated first copy of partial exon 1 and complete exons 3 to 5 of *Dmrt1* in *Pelobates cultripes* (Western spadefoot toad). Grey and white boxes represent reads aligned to the genome, showing coverage across the duplicated exons. All exons showed no expression. The RNA-seq dataset, derived from whole-body tadpole samples (SRR7817206, SRR7817207, SRR7817208, SRR7817209, SRR7817210, SRR7817211, SRR7817212, SRR7817213, SRR7817214, SRR7817215, SRR7817219, and SRR7817220), was mapped to the reference genome (*GCA_933207985.1_aPelCul1.1_chrom_genomic.fna*). The locations of each exon are shown as BED records in the bottom row. Figure S4: RNA-seq reads aligned to the duplicated second copy of exons 3 to 5 of *Dmrt1* in *Pelobates cultripes* (Western spadefoot toad). Grey and white boxes represent reads aligned to the genome, showing coverage across the duplicated exons. All exons showed no expression. The RNA-seq dataset, derived from whole-body tadpole samples (SRR7817206, SRR7817207, SRR7817208, SRR7817209, SRR7817210, SRR7817211, SRR7817212, SRR7817213, SRR7817214, SRR7817215, SRR7817219, and SRR7817220), was mapped to the reference genome (*GCA_933207985.1_aPelCul1.1_chrom_genomic.fna*). The locations of each exon are shown as BED records in the bottom row. Figure S5: RNA-seq reads aligned to *Dmrt1* in *Dendrobates tinctorius* (dyeing dart frog), showing exon-level coverage. All exons showed no read coverage. RNA-seq datasets from male brain and female liver, skin, and gut samples (SRR9304990, SRR17818134, SRR17818135, and SRR17818136) were mapped to the reference genome (*GCA_039654945.1_ASM3965494v1_genomic.fna*). The locations of each exon are shown as BED records in the bottom row. Figure S6: RNA-seq reads aligned to the partially duplicated exons 1 and 3, and the completely duplicated exon 2 of *Dmrt1* in *Dendrobates tinctorius* (dyeing dart frog), showing exon-level coverage. All duplicated exons showed no read coverage. RNA-seq datasets from male brain and female liver, skin, and gut samples (SRR9304990, SRR17818134, SRR17818135, and SRR17818136) were mapped to the reference genome (*GCA_039654945.1_ASM3965494v1_genomic.fna*). The locations of each exon are shown as BED records in the bottom row. Figure S7: RNA-seq reads aligned to *Dmrt1*, spanning exons 1 to 5 in *Rana temporaria* (European common frog). Grey boxes represent reads aligned to the genome, and thin lines connecting these boxes indicate spliced read alignments. RNA-seq datasets from male gonad samples (SRR8149028, SRR8149044, and SRR8149045) were mapped to the reference genome (*GCF_905171775.1_aRanTem1.1_genomic.fna*). The locations of each exon are shown as BED records in the top row. Figure S8: RNA-seq reads aligned to the exon 2 duplication of *Dmrt1* in *Rana temporaria* (European common frog), with no reads supporting the duplicated region. RNA-seq datasets from male gonad samples (SRR8149028, SRR8149044, and SRR8149045) were mapped to the *Rana temporaria* reference genome (*GCF_905171775.1_aRanTem1.1_genomic.fna*). The duplicated region of exon 2 is indicated as a BED track in the top row. Figure S9: RNA-seq reads aligned to exons 1 to 5 of *Dmrt1* in *Dendropsophus ebraccatus* (hourglass treefrog). Grey and white boxes represent reads aligned to the genome. The RNA-seq dataset from a female whole-body sample (SRR30172938) was mapped to the reference genome (*GCA_027789765.1_aDenEbr1.pat_genomic.fna*). The locations of each exon are shown as BED records in the bottom row. Figure S10: RNA-seq reads aligned to Dmrt1 in *Ranitomeya imitator* (imitator poison frog), showing coverage across the exons. Only exon 5 displayed very low expression, while the other exons showed no read coverage. Grey and white boxes represent reads aligned to the genome. RNA-seq datasets from brain and skin samples (ERR3169416, SRR8275032, and SRR29319291) were mapped to the reference genome (GCA_032444005.1_aRanImi1.pri_genomic.fna). The locations of each exon are shown as BED records in the bottom row. Figure S11: RNA-seq reads aligned to the duplicated exon 2 of *Dmrt1* in *Ranitomeya imitator* (imitator poison frog), showing no read coverage. RNA-seq datasets from brain and skin samples (ERR3169416, SRR8275032, and SRR29319291) were mapped to the reference genome (*GCA_032444005.1_aRanImi1.pri_genomic.fna*). The locations of each exon are shown as BED records in the bottom row. Figure S12: *Dmrt1* gene expression (in FPKM) across 12 anurans. Gene expression levels were calculated using Cufflinks. Figure S13: *Dmrt1* gene expression (in FPKM) across 2 anurans. Gene expression levels were calculated using Cufflinks. Figure S14: *d_N_*: *d_S_*, and ω (*d_N_/d_S_*) for each species based on free-ratio models across 53 anuran species. Figure S15: Plot of *d_S_* values of *Dmrt1* and its duplicated copies for 53 anurans, including the status of sex chromosomes reported in the literature. Boxplot of *d_S_* values. Figure S16: Alignment of *Dmrt1* protein sequences (amino acid positions 1–191), visualized using Jalview. Figure S17: Alignment of *Dmrt1* protein sequences (amino acid positions 190–370), visualized using Jalview. Supplementary tables: Table S1: Genome assemblies from NCBI for 54 amphibian species. Table S2: Available raw genomic sequencing reads and the NCBI SRA number. Table S3: RNAseq raw reads data from NCBI, and the related information on tissue, sex and developmental stage. Table S4: Assess the completeness of genome/transcriptome assemblies using BUSCO. Table S5: Exons of *Dmrt1* and their lengths. Table S6: RNAseq data used for expression analysis between males and females, for species with at least one tissue in both sexes. Table S7: The intact and duplicated copies of *Dmrt1* in 8 anurans, along with their locations and lengths. Table S8: *Dmrt1* gene synteny for annotated genomes of 12 species and BLAST hit synteny for chromosome-level genomes. Table S9: *Foxl2* gene synteny for annotated genomes of 12 species. Table S10: *Sox3* gene synteny for annotated genomes of 12 species and BLAST hit synteny for chromosome-level genomes. Table S11: *Bod1l* gene synteny for annotated genomes of 12 species and BLAST hit synteny for chromosome-level genomes. Table S12: (A) Omega, *d_N_* and *d_S_* from the branch test for 54 species of *Dmrt1* with *DmW* and *Pelodytes ibericus* duplicated copy of *Dmrt1* gene; (B) Omega, *d_N_* and *d_S_* from the free-ratio model for 54 species of *Dmrt1* with *DmW* and *Pelodytes ibericus* duplicated copy of *Dmrt1* gene; (C) Branch test for selection in 54 species of *Dmrt1* with *DmW* and *Pelodytes ibericus* duplicated copy of *Dmrt1* gene; (D) Branch-site test for 54 species of *Dmrt1* with *DmW* and *Pelodytes ibericus* duplicated copy of *Dmrt1* gene. Table S13: (A) Omega, *d_N_* and *d_S_* from the branch test for 52 species of *Foxl2*; (B) Branch test for selection in 52 species of *Foxl2*. Table S14: (A) Omega, *d_N_* and *d_S_* from the branch test for 53 species of *Sox3*; (B) Branch test for selection in 53 species of *Sox3*.
